# Cochlear Implantation Following Neurobrucellosis-Related Bilateral Profound Sensorineural Hearing Loss: A Case Report and Literature Review

**DOI:** 10.7759/cureus.110831

**Published:** 2026-06-14

**Authors:** Ozan Özdemir, Ismail Kaygisiz, Emre Baran, Ramazan Celik

**Affiliations:** 1 Otolaryngology - Head and Neck Surgery, University of Health Sciences Istanbul Training and Research Hospital, Istanbul, TUR

**Keywords:** brucellosis, cochlear implantation, labyrinthitis ossificans, neurobrucellosis, sensorineural hearing loss

## Abstract

Brucellosis is the most common bacterial zoonosis worldwide, and neurological involvement - neurobrucellosis - can cause bilateral profound sensorineural hearing loss (SNHL) that does not respond to antibiotic therapy. We report cochlear implantation (CI) outcomes in a 60-year-old man with confirmed neurobrucellosis who developed bilateral profound SNHL that provided no functional benefit after a six-month binaural hearing aid trial. Preoperative temporal bone computed tomography and magnetic resonance imaging confirmed intact cochlear anatomy with no labyrinthitis ossificans on either modality. Right-ear CI was performed after an infectious disease consultation confirmed sustained disease remission. At 12-month follow-up, sound-field thresholds were 40 dB hearing level (HL) at 500 Hz and 45-50 dB HL at 1,000-4,000 Hz. The patient was able to follow face-to-face conversations without lip-reading. We also review all previously published cases in which CI was performed or considered in this setting, highlighting the consistent absence of labyrinthitis ossificans, unlike most other bacterial causes of deafness, which facilitates full electrode insertion and supports favorable outcomes. CI is safe and effective for neurobrucellosis patients with bilateral profound SNHL, and confirmed disease remission before surgery is a mandatory prerequisite.

## Introduction

Brucellosis is the most common bacterial zoonosis worldwide, with approximately 500,000 new cases annually. The World Health Organization classifies it as a neglected disease disproportionately affecting populations in the Mediterranean basin, the Middle East, Central Asia, sub-Saharan Africa, and Latin America [1]. In its active phase, brucellosis typically presents with undulant fever, night sweats, fatigue, arthralgia, hepatosplenomegaly, and lymphadenopathy; these systemic features may precede or accompany organ-specific complications. Neurological involvement-neurobrucellosis-complicates 1.7%-10% of cases and may manifest as meningitis, meningoencephalitis, myelitis, radiculopathy, or cranial neuropathy [2,3]. The vestibulocochlear nerve is the cranial nerve most commonly affected: Gul et al.'s pooled analysis of 187 neurobrucellosis patients documented eighth-nerve involvement in 19% [2], and Bayazit et al. described a characteristic high-frequency loss pattern that can progress to bilateral profound sensorineural hearing loss (SNHL) despite adequate antimicrobial therapy [4]. Once hearing loss reaches this degree and fails to respond to amplification, cochlear implantation (CI) becomes the most effective rehabilitation strategy. Only a handful of such cases have been described [5-13], and evidence-based guidance remains absent. We report our experience with CI in a 60-year-old man with neurobrucellosis-related bilateral profound SNHL and review all published cases.

## Case presentation

Written informed consent was obtained from the patient for the publication of this case report and all accompanying clinical images, in accordance with the Declaration of Helsinki. No institutional review board approval was required at our institution for a single de-identified case report.

A 60-year-old man presented with a two-year history of progressive bilateral hearing loss. He reported no tinnitus, dizziness, or neurological symptoms, and had no prior history of neurological disease.

Two years before presentation, he had been hospitalized with a severe headache and altered consciousness. A Brucella tube agglutination test was strongly positive (titer 1:640) and confirmed by enzyme-linked immunosorbent assay (ELISA), establishing neurobrucellosis. Triple antibiotic therapy with doxycycline, rifampicin, and ceftriaxone was administered under infectious disease supervision. Microbiological remission was achieved, but bilateral hearing deteriorated progressively over the following year.

Audiometric evaluation revealed bilateral profound SNHL: speech reception threshold (SRT) was 100 dB hearing level (HL) (right) and 95 dB HL (left); speech discrimination was unobtainable. Tympanometry showed bilateral type A curves; acoustic reflexes and distortion-product otoacoustic emissions (DPOAEs) were absent bilaterally. A six-month binaural hearing aid trial offered no meaningful benefit, with aided SRT remaining at 75 dB HL. Audiometric results are shown in Figure 1.

**Figure 1:**
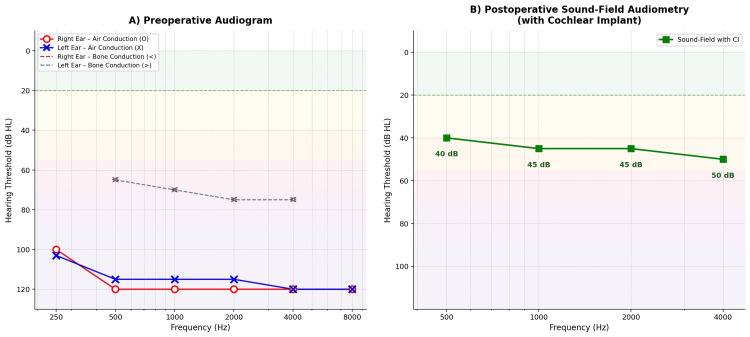
Audiometric results. (A) Preoperative pure-tone audiometry demonstrating bilateral profound sensorineural hearing loss (SNHL); air conduction (right ear: circles; left ear: crosses) and bone conduction thresholds (right: <; left: >) are shown. (B) Cochlear implant-aided sound-field thresholds at 12-month post-activation follow-up.

High-resolution temporal bone computed tomography (CT) and internal auditory canal magnetic resonance imaging (MRI) confirmed intact cochlear anatomy, bilaterally patent scala tympani and scala vestibuli, normal cochlear fluid signal, and preserved cochlear nerve caliber, with no labyrinthitis ossificans (LO) on either modality (Figure 2). There was no meningeal enhancement or intracranial pathology.

**Figure 2:**
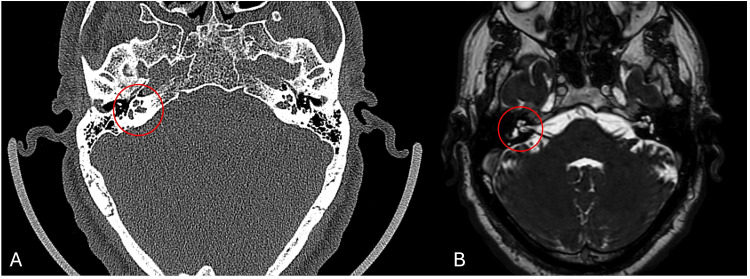
Preoperative imaging of the right temporal bone. (A) Axial high-resolution computed tomography demonstrating patent scala tympani and scala vestibuli with no evidence of labyrinthitis ossificans (red circle). (B) Axial T2-weighted magnetic resonance imaging showing normal high-signal cochlear fluid with preserved scala architecture and no evidence of ossification or fibrosis (red circle).

Following multidisciplinary team review (otolaryngologist, audiologist, radiologist, and psychologist) and infectious disease specialist confirmation of sustained brucellosis remission, right-ear CI was performed via a postauricular transmastoid approach. A 28-mm lateral-wall electrode array (MED-EL Synchrony FLEX 28; MED-EL Medical Electronics, Innsbruck, Austria) was inserted through the round window membrane without resistance, achieving atraumatic full-length insertion. Intraoperative neural response telemetry and stapedial reflex testing confirmed responses at all electrode contacts. Postoperative radiographs confirmed correct intracochlear electrode placement (Figure 3). The patient was discharged on postoperative day three without complications.

**Figure 3:**
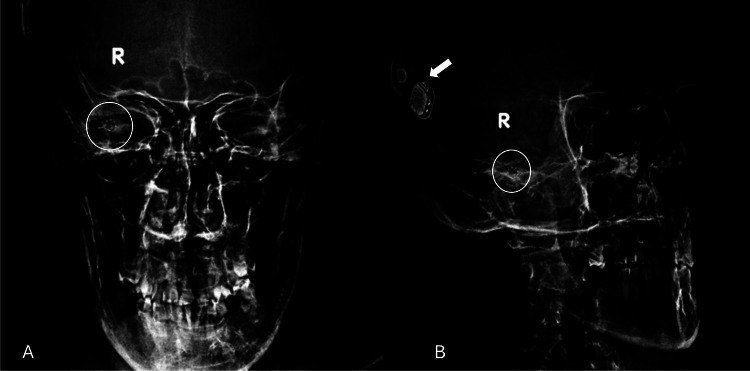
Postoperative radiographs confirming correct intracochlear electrode placement. (A) Stenvers view showing the intracochlear electrode array (white circle). (B) Schuller (lateral) view showing the receiver-stimulator unit (white arrow) and the intracochlear electrode array (white circle).

The device was activated one month postoperatively. At 12-month follow-up, sound-field thresholds were 40 dB HL at 500 Hz, 45 dB HL at 1,000 and 2,000 Hz, and 50 dB HL at 4,000 Hz. The patient followed face-to-face conversations without lip reading, though noisy environments remained challenging, as expected at this stage of adaptation.

## Discussion

Neurobrucellosis-related SNHL is typically bilateral, progressive, and irreversible. Proposed mechanisms include direct Brucella invasion of the labyrinth with outer hair cell destruction, immune-mediated vestibulocochlear neuropathy (including anti-GM1 ganglioside antibody formation), demyelination of central auditory pathways, endotoxin-mediated vascular spasm causing avascular cochlear injury, and small-vessel vasculitis [2,5,6]. Messaoudi et al. reported a clinically important finding: auditory evoked potential testing confirmed preserved retrocochlear and central auditory pathway function despite severe bilateral SNHL [7]. Because intact retrocochlear function is an essential prerequisite for successful CI, neurobrucellosis patients may satisfy this criterion more consistently than the severity of their hearing loss would suggest.

The most surgically significant feature of neurobrucellosis-related SNHL is the consistent absence of LO across all published cases (Table 1). In other forms of bacterial meningitis - particularly pneumococcal - LO develops in up to 30% of patients, can prevent complete electrode insertion, and demands prompt surgery before ossification advances [12]. To date, no published case of neurobrucellosis-related hearing loss has reported LO on preoperative imaging [5,7-11]. This likely reflects the predominantly granulomatous and lymphocytic inflammatory response induced by Brucella, in contrast to the fibrinopurulent exudates that calcify the cochlear lumen in other bacterial meningitides [14]. In our patient, the electrode advanced the full 28 mm through the round window without intraluminal resistance - a direct reflection of this pathobiological distinction.

**Table 1:** Summary of published neurobrucellosis cases with sensorineural hearing loss evaluated for or receiving cochlear implantation. AB = Advanced Bionics; F = female; LO = labyrinthitis ossificans; M = male; N/R = not reported; SF = sound-field threshold (dB HL)

Author, Year	n	Age/Sex	Electrode Used	Hearing Outcome	LO	Follow-Up
Guneri et al., 2009 [8]	1	32/M	Nucleus Contour Advance (24-ch, perimodiolar)	SF within normal limits	None	18 months
Bajin et al., 2016 [9]	1	35/F	Nucleus Contour Advance (perimodiolar)	Successful auditory gain	None	24 months
Alshalan et al., 2022 [10]	2	52/F; 48/F	AB HiFocus Mid-Scala (perimodiolar)	Case 1: SF 15 dB HL; Case 2: SF 25 dB HL	None	N/R
Ocak et al., 2015 [11]	1	54/F	CI512 Contour Advance (perimodiolar)	SF 40 dB HL	None	12-15 months
Present case	1	60/M	28-mm lateral-wall array (round window insertion)	SF 40-50 dB HL	None	12 months

Table 1 summarizes all reported neurobrucellosis cases in which CI was performed or considered. Four groups have described implanted patients [8-11]; additional patients have been identified as candidates [7] or referred for evaluation [5] without proceeding to implantation. In every case, including our own, no LO was encountered, no surgical complications occurred, and all implanted patients achieved sound-field thresholds in the moderate or better range. Across all reported implanted cases (n = 6), postoperative sound-field thresholds ranged from 15 to 50 dB HL over follow-up periods of 12 to 24 months, with no instances of LO or incomplete electrode insertion. Our patient's 40-50 dB HL result at 12 months is consistent with previously reported outcomes.

Round window insertion was chosen in preference to cochleostomy because it avoids drilling at the basal hook region, preserves residual cochlear architecture, and reduces the risk of intracochlear trauma-advantages particularly relevant when residual nerve fiber integrity may contribute to CI performance. The electrode traveled the full cochlear length without resistance, confirming the patent intracochlear anatomy characteristic of neurobrucellosis.

Two prerequisites governed our clinical decision-making. First, confirmed disease remission before surgery: active brucellosis would preclude implantation and risk device-related infectious complications; specialist consultation before any surgical planning is therefore mandatory. Second, timely implantation to minimize auditory deprivation: the approximately two-year interval in our patient is suboptimal, but adults with postlingual profound deafness can still achieve meaningful CI benefit after several years because auditory memory and residual cortical plasticity are preserved [9]. Our patient's ability to follow conversations without lip reading at 12 months supports this.

The main limitations of this report are those inherent to any single case: the findings cannot be generalized, and causal conclusions cannot be drawn. Notably, preoperative auditory brainstem response (ABR) testing and cerebrospinal fluid analysis were not available; ABR data would have helped clarify whether the hearing loss was primarily cochlear, neural, or mixed, and CSF findings would have contributed to pathophysiological characterization. Additionally, our follow-up was limited to a single 12-month assessment without serial standardized speech perception testing at earlier intervals. Prospective multicenter studies incorporating ABR, electrophysiological protocols, and serial outcome measures are needed to guide CI decision-making in this population, though the rarity of the condition makes such studies challenging to organize.

## Conclusions

Neurobrucellosis-related bilateral profound SNHL is rare but permanently disabling, and antibiotic treatment does not reverse it once established. For patients who derive no benefit from hearing aids, CI is safe, feasible, and effective. The consistent absence of LO - unlike most bacterial causes of deafness - creates favorable surgical conditions that permit full electrode insertion and support good outcomes. Pre-implant confirmation of disease remission and timely surgery are the two most important modifiable management factors. We advocate for early and proactive CI consideration in neurobrucellosis patients with disabling bilateral profound SNHL.
